# Bio-compatible organic humidity sensor based on natural inner egg shell membrane with multilayer crosslinked fiber structure

**DOI:** 10.1038/s41598-019-42337-0

**Published:** 2019-04-09

**Authors:** Muhammad Umair Khan, Gul Hassan, Jinho Bae

**Affiliations:** Department of Ocean System Engineering, Jeju National University, 102 Jejudaehakro, Jeju, 63243 Republic of Korea

## Abstract

In this paper, we propose a novel bio-compatible organic humidity sensor based on natural inner egg shell membrane (IESM) with multilayer cross linked fiber structure that can be used as a substrate as well as a sensing active layer. To fabricate the proposed sensors, two different size inter digital electrodes (IDEs) with 10 mm × 4 mm for sensor 1 and 12 mm × 6 mm for sensor 2 are printed on the surface of the IESM through Fujifilm Dimatix DMP 3000 inkjet material printing setup, which have finger width of 100 μm and space of 100 μm. The fabricated sensors stably operates in a relative humidity (RH) range between 0% RH to 90% RH, and its output impedance and capacitance response are recorded at 1 kHz and 10 kHz. The response time (T_res_) and recovery time (T_rec_) of sensor 1 are detected as ~1.99 sec and ~8.76 sec, respectively and the T_res_ and T_rec_ of sensor 2 are recorded as ~2.32 sec and ~9.21 sec, respectively. As the IESM for the humidity sensor, the natural materials can be implemented in our daily life as they open a new gate way for bio-compatible devices.

## Introduction

In environment sensing, there are various factors to be detected like humidity level^1^, temperature^2^, various gases (N_2_, CO_2,_ O_2_, etc.)^3,4^, and light intensity^5^. In these features, a humidity sensing is widely investigated by many researchers^6–8^. Sensors based on impedance and capacitance responses are the best choice for humidity sensing due to low cost, easy fabrication, and nominal time response^9^, which can detect amount of water content in environment with following parameters like impedance^10^, capacitance^11^ and resistance^12–14^. To fabricate a humidity sensor, the various materials are studied like, graphene^15^, organic^16^, inorganic^17^, organic-inorganic nano-composite^18^, and other composites^19,20^.

For an eco-friendly, low cost and high performance devices, many researchers are exploring natural materials, for example, humidity sensing layer and substrate using onion membrane^21^, piezoelectric nanogenerator using onion skin^22^, silk fiber applied in electronic devices and non-volatile resistive memory^23,24^, and fish scale based piezoelectric nanogenerator^25^. Hence, a biomaterial can be obtained from a hen egg and it is widely using as food in the world. It can be applied in different applications like egg albumen as an active layer for non-volatile resistive memory^26^, a high performance electrode for super capacitor using carbonized egg shell^27^, a bio-sensor to determine the glucose using inner egg shell membrane (IESM)^28^, and piezoelectric flexible nanogenerator using IESM^29^. The self-assembled nano fiber humidity sensor (based on donor and acceptor mechanism) can be used for monitoring purpose^30^. Although the IESM is structured with natural multilayer cross linked fibers, it is not applied for a humidity sensor. The natural biomaterials available in our daily life have potential to be employed in sensing applications as they can opens a new possibilities towards bio-compatible electronic devices.

Many researchers are focusing on transferable, wearable, and implantable electronic devices on thin substrates, which are highly flexible and light in weight^31^. Moreover, such substrates are used only to support device printed on them with no sensing properties and additional sensing layer is required to be top printed on electrodes. For these attributes, natural biological organic materials available in our daily life are very attractive for sensing in wearable electronic devices^22^. In this expectation, to achieve good results, the various printing methods are utilizing for high performance and transferable wearable devices^32,33^. The conventional printing methods utilized to transfer wearable electronic devices are very complex and damage may occur while transferring to arbitrary shaped substrate^31^. While an IESM is ultra-thin with thickness of ~19 μm, it is allowed to transfer to any arbitrary shaped substrate. The IESM can be transfer in a single step to an arbitrary shape substrate without causing any damage.

**Figure 1:**
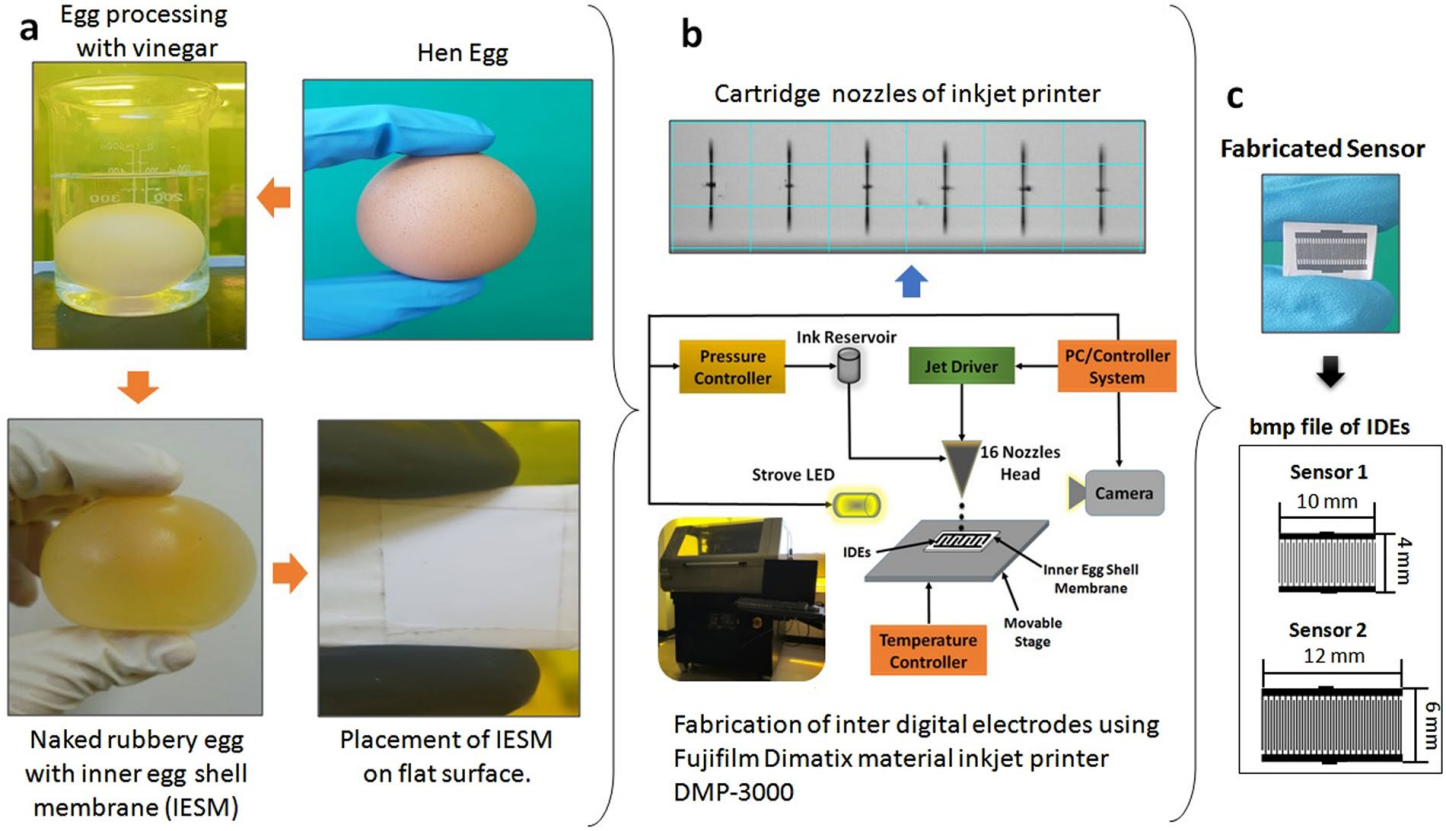
(**a**) The Processing of the IESM. (**b**) The wave shape of printing nozzles of the Fujifilm DMP-3000 inkjet printer. (**c**) Dimension of the IDEs and Printed IDEs on the surface of the IESM using inkjet printer.

The proposed humidity sensor is fabricated by using the IESM as an active sensing layer and substrate. The IESM is contacted with the egg albumen and an external contact with the egg shell minerals^34,35^. Especially, the IESM has fibrous and porous structure, which is a suitable to absorb a permeable solvent like water; hence it can be used as a humidity sensing active layer. To get a signal from the IESM, we fabricated inter digital electrodes (IDEs) pattern with finger width of 100 μm and spacing of 100 μm through Fujifilm Dimatix DMP-3000 material as shown in Fig. 1. We fabricated two sensors (sensor 1 and sensor 2), and their size are 10 mm × 4 mm for sensor 1 and 12 mm × 6 mm for sensor 2. The fabricated sensor is tested with homemade humidity sensing setup, which has a impedance and capacitance response range from 0% RH to 90% RH, and this bio-compatible humidity sensor achieves a response time (T_res_) of ~1.99 sec and a recovery time (T_rec_) of ~8.76 sec using sensor 1 and T_res_ of ~2.32 sec and T_rec_ of ~9.21 sec using sensor 2. The surface morphology and cross sectional image of the proposed sensor is analyzed with NV-2000 Universal non-contact surface profiler, Scanning electron microscopy (SEM) Jeol JSM-7600F and TESCAN MIRA 3 scanning transmission electron microscope (STEM), and Fourier transform infrared spectroscopy (FTIR) is performed with Bruker IFS 66 V spectrometer. To find the material characteristics of the IESM, its elemental composition is performed with energy dispersive X-ray (EDS) spectrometer.

## Results and Discussion

### SEM of IESM and IDEs

**Figure 2:**
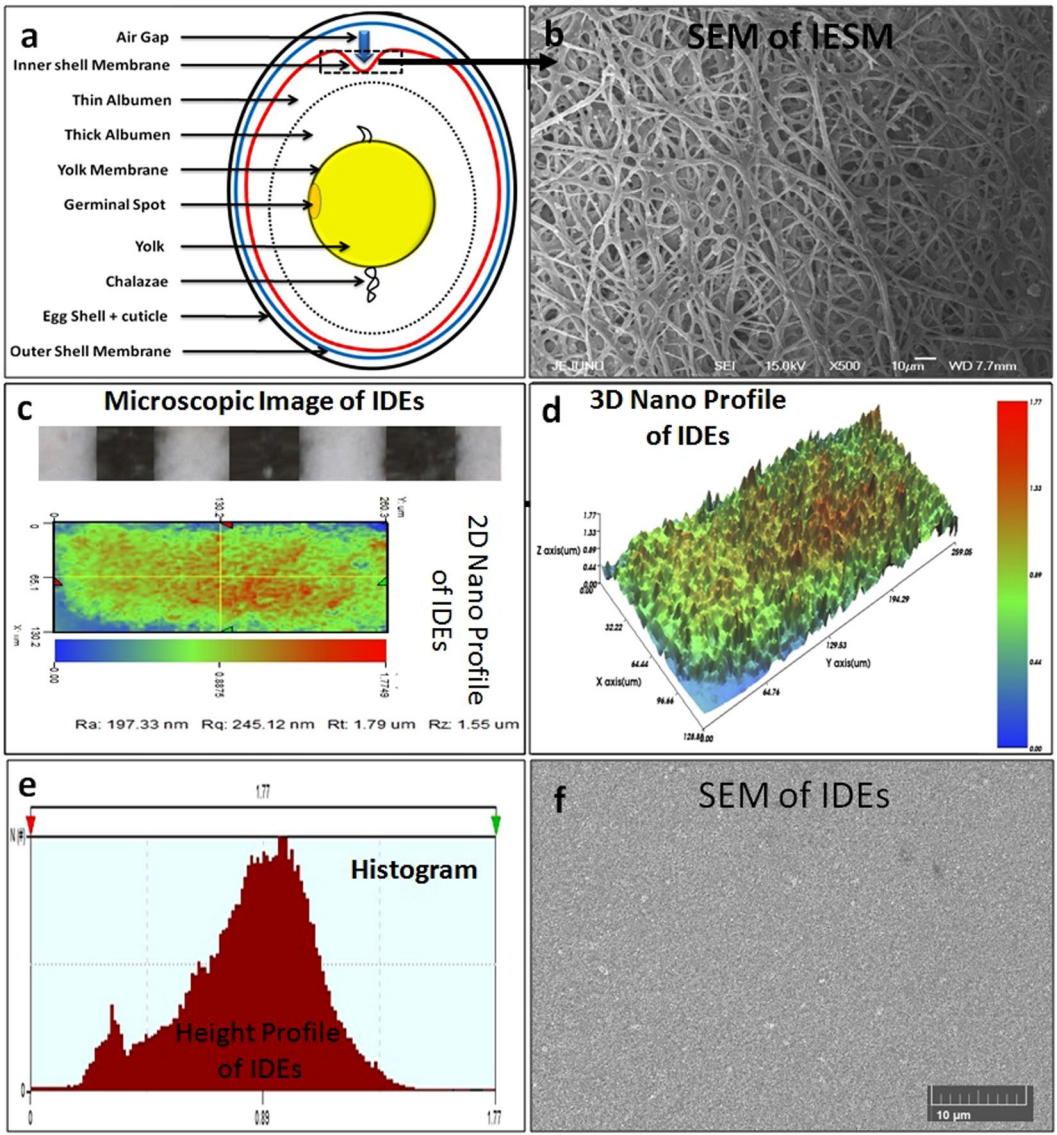
(**a**) Structural image of hen egg. (**b**) FESEM image of IESM. (**c**) Microscopic image of printed IDEs showing electrode width of 100 μm and electrode spacing of 100 μm. 2D surface roughness of 197.33 nm and (**d**) 3D surface nano profile with thickness of 1.77 μm. (**e**) Histogram of height for nano profile of IDEs. (**f**) SEM image of the IDEs.

**Figure 3:**
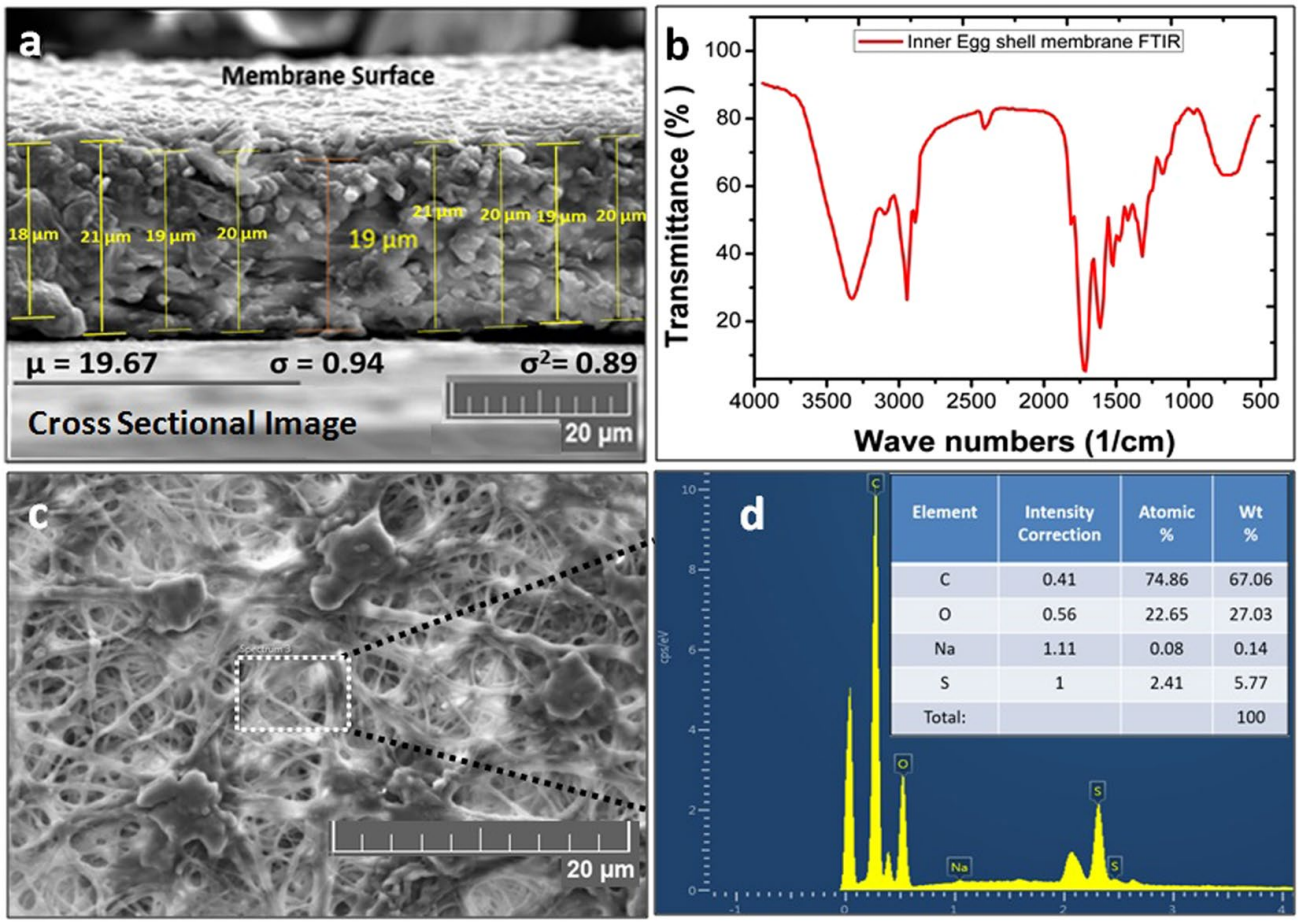
(**a**) Cross sectional image of IESM. (**b**) FTIR of the IESM. (**c**) Surface morphology of the IESM using TESCAN MIRA 3. (**d**) The EDS representation of the IESM and insert image showing element composition of the IESM.

Hen egg is shown in Fig. 2a, which insure that IESM is present between egg albumen and egg shell. The IESM is consisted of an intricate lattice network of fibers^36^. A network-like structure is perceived in the natural IESM with 10 μm scale at ×500 magnification as shown in Fig. 2b. This indicates that the IESM is consisted of cavities and pores of diameter 5 μm, and has highly crosslinked fibers of protein with diameter 0.5–1.5 μm. Figure 2c shows the microscopic image of the patterned IDEs. The 2D and 3D nanoprofile of the IDEs is illustrate as shown in Fig. 2c,d, which insures that electrodes are properly fabricated with the inkjet printer. Figure 2f shows the surface morphology of IDEs with magnification level of 10 µm, which insures that the electrodes are properly fabricated on the surface of IESM. The cross sectional of an IESM is shown in Fig. 3a with magnification level of 20 μm, which indicate that dried IESM have thickness of ~19 μm. The variability of the thickness of dried IESM is presented in Fig. 3a with mean value (µ) of 19.67 µm, standard deviation (σ) of 0.94 µm and variance (σ^2^) of 0.89 µm. These results shows that the thickness of the dried IESM is almost uniform with small value of standard deviation as shown in Fig. 3a.

### 2D and 3D Nano profile of IDEs

The 2D and 3D nano profile of the printed IDEs is shown in Fig. 2c and d, which indicates that printed electrodes have roughness of 197.33 nm and the IDEs are uniformly fabricated. The height profile of IDEs is shown in Fig. 2e, which indicate that average height of the Ag nanoparticle based IDEs are ~1.77 μm.

### FTIR of IESM

The Fourier transform infrared spectroscopy-potassium bromide (FTIR-KBr) spectra of IESM showed the characteristic absorption peaks related to the organic structure of the membrane. In the FTIR spectra of an IESM shown in Fig. 3b, the amide and protein characteristic peaks are found as three highly intense peaks. Amide I shows two sharper bands of C=O stretching vibrations at 1650 cm^−1^, amide II shows NH in plane bending and CN stretching at 1440 cm^−1^, and amide A shows NH stretching vibration at 3400 cm^−1^. An absorption band shows stretching vibration of C-S bonds appeared at 670 cm^−1^, which is associated with cysteine-rich proteins of an IESM fibers^36,37^. A sharp absorption peak of eggshell was also present at 875 cm^−1^ which shows out of the plane bending vibration of (CO_3_^−2^)^38^.

### EDS mapping of IESM

**Figure 4:**
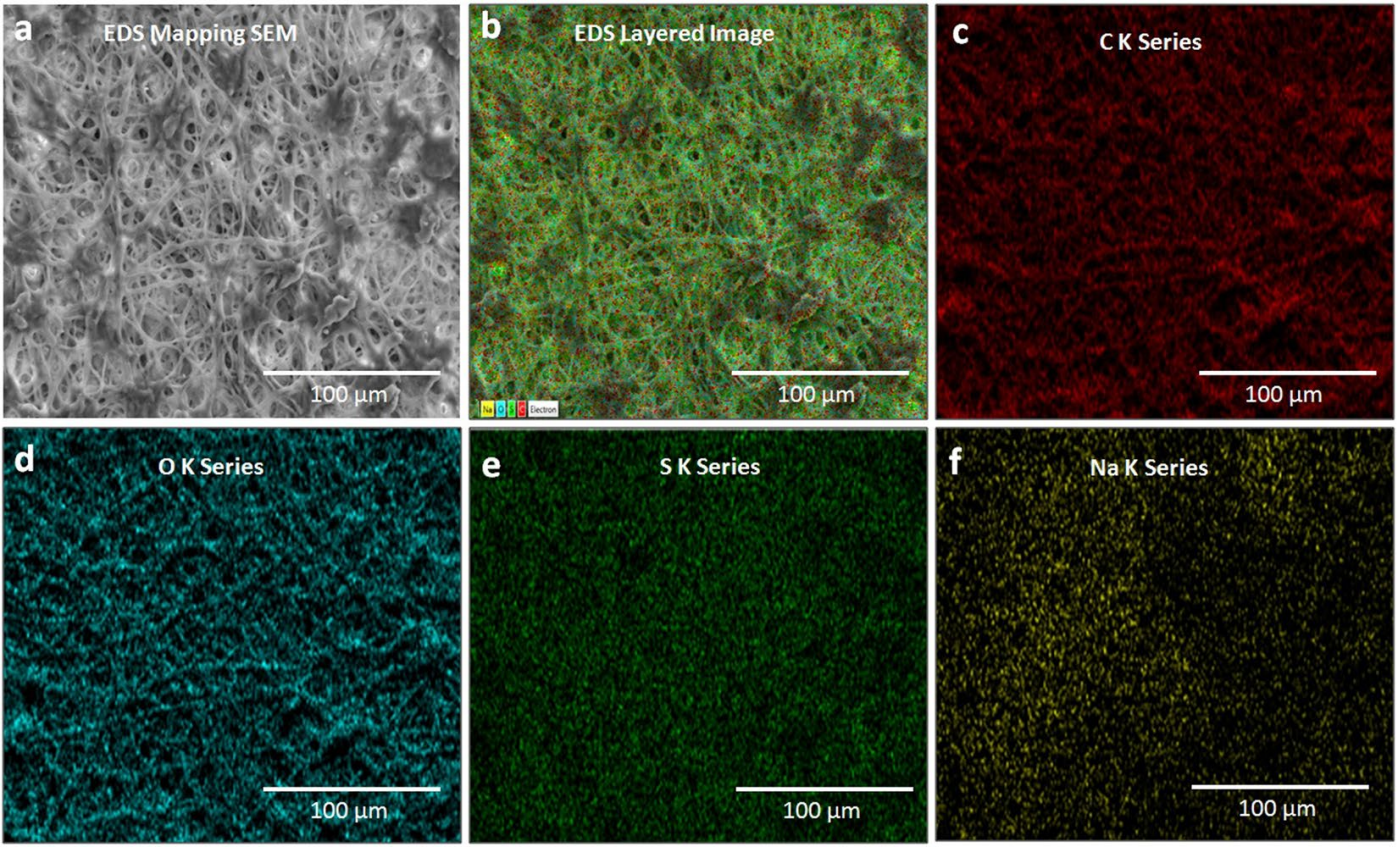
(**a**) EDS mapping SEM image of IES. (**b**) EDS layer image, and element mapping of the IESM representing as (**c**) C K series, (**d**) O K series, (**e**) S K series, and (**f**) Na K series.

The elemental composition on the surface of IESM can be confirmed by energy dispersive X-ray spectroscopy (EDS) as depicted in Fig. 3c with magnification of 20 μm. The EDS energy dispersive analysis of X-ray spot profile of the IESM clearly shows peaks of C, O, S and Na as inset shown of Fig. 3d. EDS mapping of the IESM is performed with TESCAN MIRA 3. As EDS SEM image is shown in Fig. 4a, it shows the magnification level of 100 µm and Fig. 4b shows the EDS layered image which insurances the presence of carbon series with weight 67.06%, Oxygen series with weight 27.03%, Sulfur series with weight 5.77%, and Sodium series with weight 0.14% as shown in Fig. 4c–f. EDS confirms the presence of carbonyl, amino protein and carboxyl group as we discussed in FTIR of IESM.

### Impedance response

**Figure 5:**
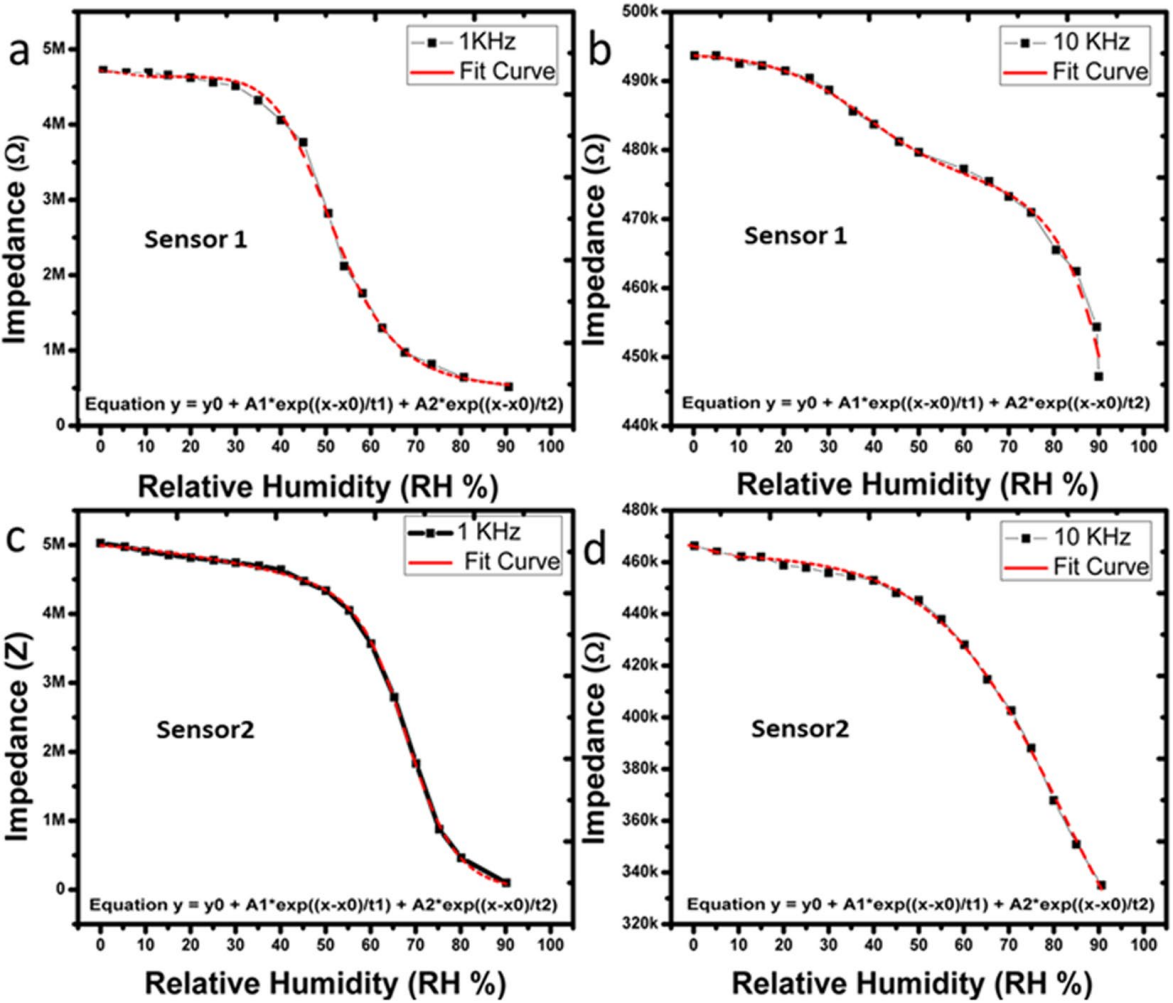
Impedance response of the sensor 1 at (**a**) 1 kHz and (**b**) 10 kHz. Impedance response of the sensor 2 at (**c**) 1 kHz and (**d**) 10 kHz.

The impedance response of the humidity sensor was recorded on different levels of humidity in the environment. The effect of humidity on the surface of the IESM was recorded on different frequencies as shown in Fig. 5. These results clearly indicate that the impedance of Sensor under test (SUT) is decreased with increase in humidity level. This behavior is consistent at 1 kHz and 10 kHz of sensor 1 and sensor 2 as shown in Fig. 5a–d. The increase in test frequency results in the decrease in the impedance state of the SUT. It indicates that the impedance state of the SUT is inversely proportional to the test frequency as given by Eq. (1). Here, R, *f*, and *c* represent the resistance, test frequency, and capacitance of SUT, respectively.

1 $$Z=\frac{1}{2\pi fc}+R\,$$

### IESM mounted on arbitrary surfaces

**Figure 6:**
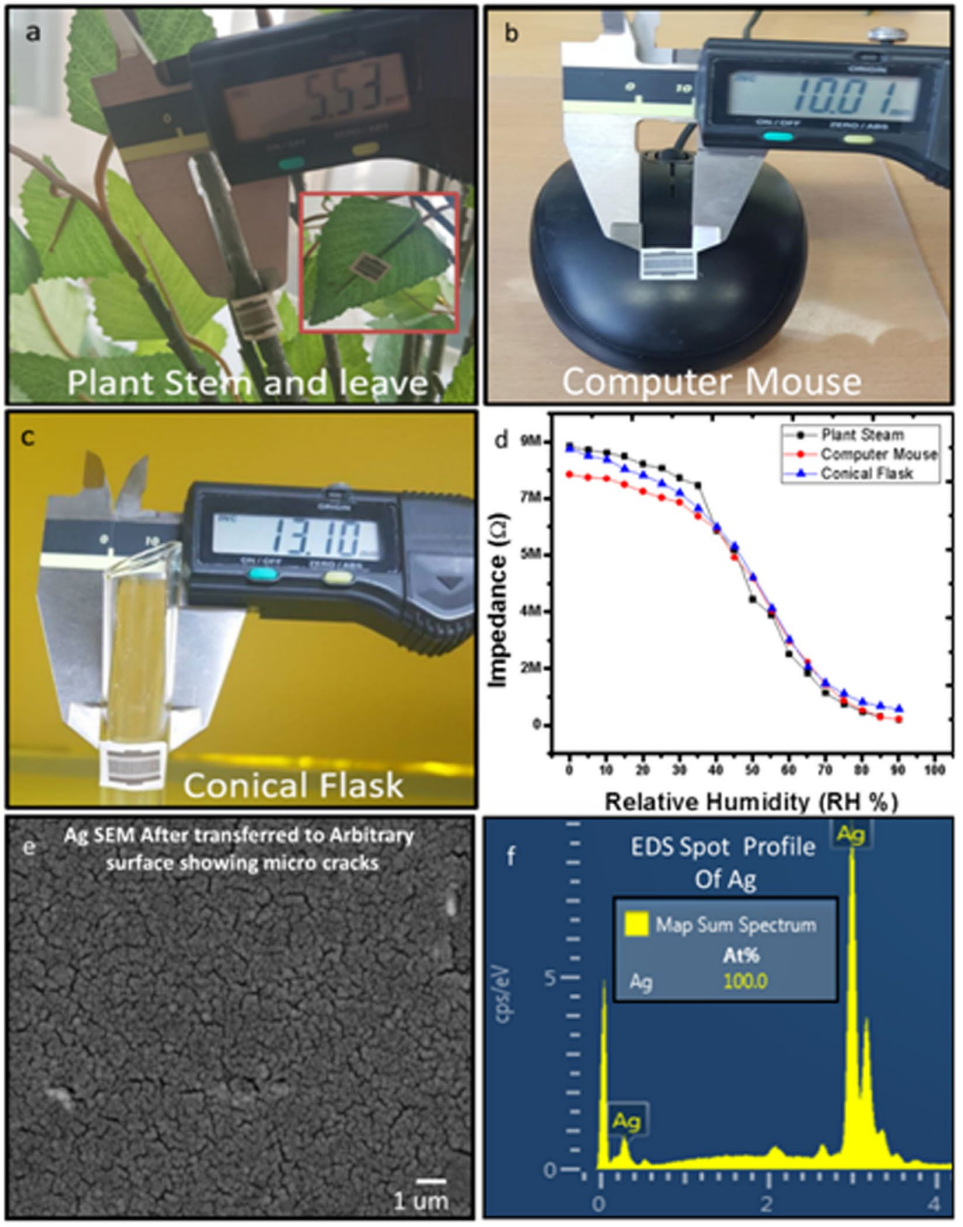
Images of the fabricated sensor on IESM transferred to various shaped as a substrates: (**a**) plant stem as a substrate with 5.53 mm diameter, (**b**) computer mouse with diameter 10 mm, and (**c**) round shape conical flask with diameter 13.1 mm. (**d**) Impedance response of IESM on transferred to arbitrary surface. (**e**) FESEM of IDEs after transferred to arbitrary surface. (**f**) EDS spot profile shows the presence of Ag in FESEM.

The fabricated sensor was mounted/transferred on the different arbitrary surfaces as a substrate. The target substrates have different uneven surfaces with different curvature, which includes plant leaf and stem, computer mouse, and conical flask as shown in Fig. 6a–c. The SUT is attached to different targeted surfaces with different curvature are shown in Fig. 6a–c. The output response of SUT shown in Fig. 6d is obtained from the transferred on plant steam, computer mouse and conical flask. The impedance response of the sensor was increased due to the formation of micro cracks in IDEs after bending as shown in Fig. 6e. Figure 6 indicates that IESM can easily be transferred to an arbitrary shape substrate without causing any damage. The EDS spot profile of the IDEs after transferred to arbitrary surface is shown in Fig. 6f.

### Capacitance response

**Figure 7:**
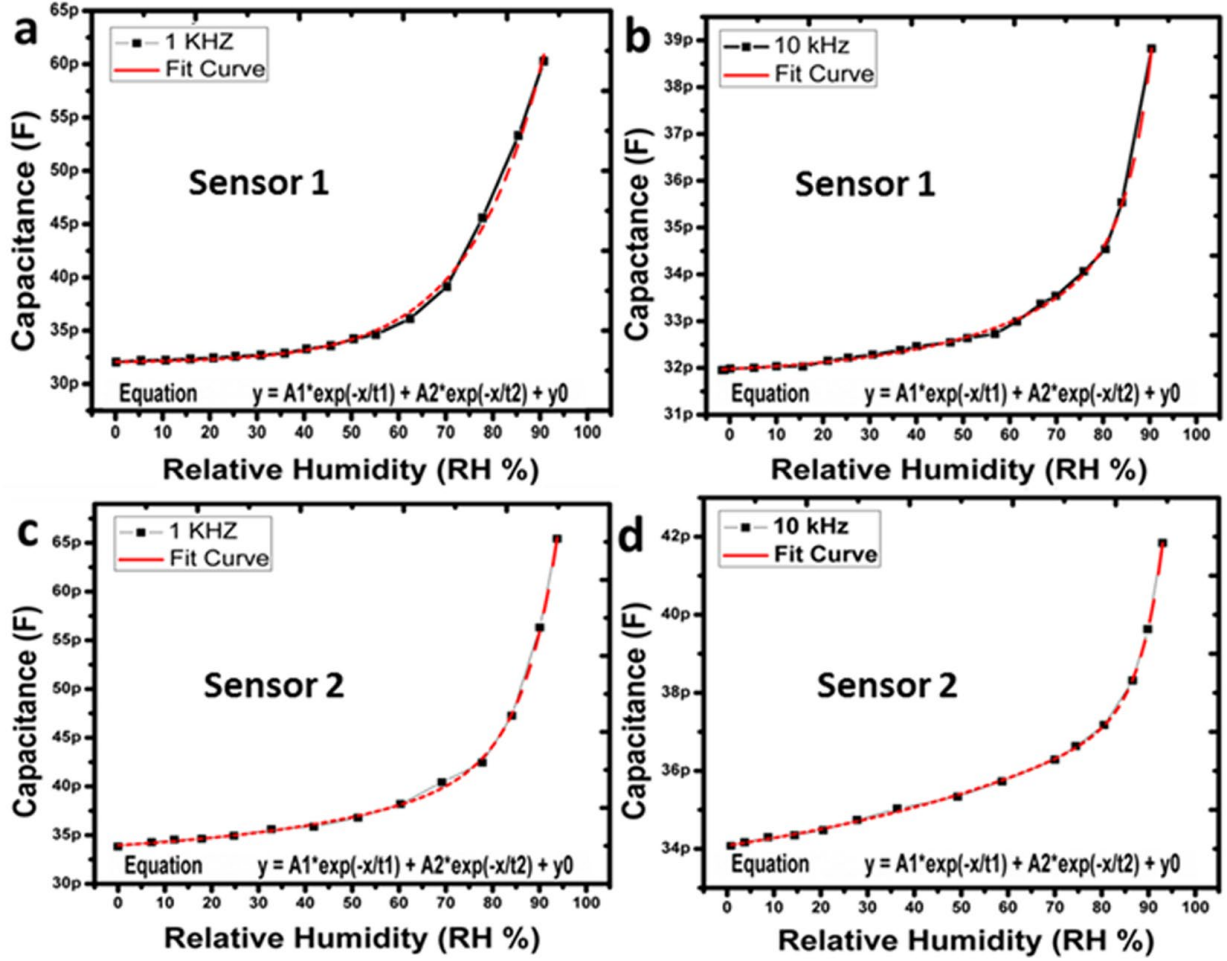
Capacitance response of the sensor 1 at (**a**) 1 kHz and (**b**) 10 kHz. Capacitance response of the sensor 2 at (**c**) 1 kHz and (**d**) 10 kHz.

As shown in Fig. 7, the absorption of water molecules changes the dielectric coefficient, as a result change in capacitance of SUT. The capacitance of SUT increases with decrease in impedance with increase in relative humidity. The change in capacitance can be observed on different test frequencies of sensor 1 and sensor 2 as given in Fig. 7a–d. The intrinsic capacitance of SUT decreases with increase in test frequency range from 1 kHz to 10 kHz. This phenomenon is due to flow of leakage conduction (*γ*) and capacitance at higher frequencies. The capacitance of SUT with leakage conduction is given in Eq. (2).

2 $$C={\varepsilon }^{\ast }\,{C}^{o}=({\varepsilon }_{r}{\textstyle \text{-}}\,i\frac{\gamma }{2\pi f{\varepsilon }_{o}}){C}_{o}$$

where, *ε*, *C*_0_, *γ*, *f*, *ε*_*r*_, and *ε*_0_ are the complex dielectric contact, expected capacitance, conductance, frequency, relative dielectric constant of ideal capacitor, and permittivity of free space, respectively. The capacitance response of the SUT ensures that it can be implemented in real time systems without any extra circuit.

### Transient response

**Figure 8:**
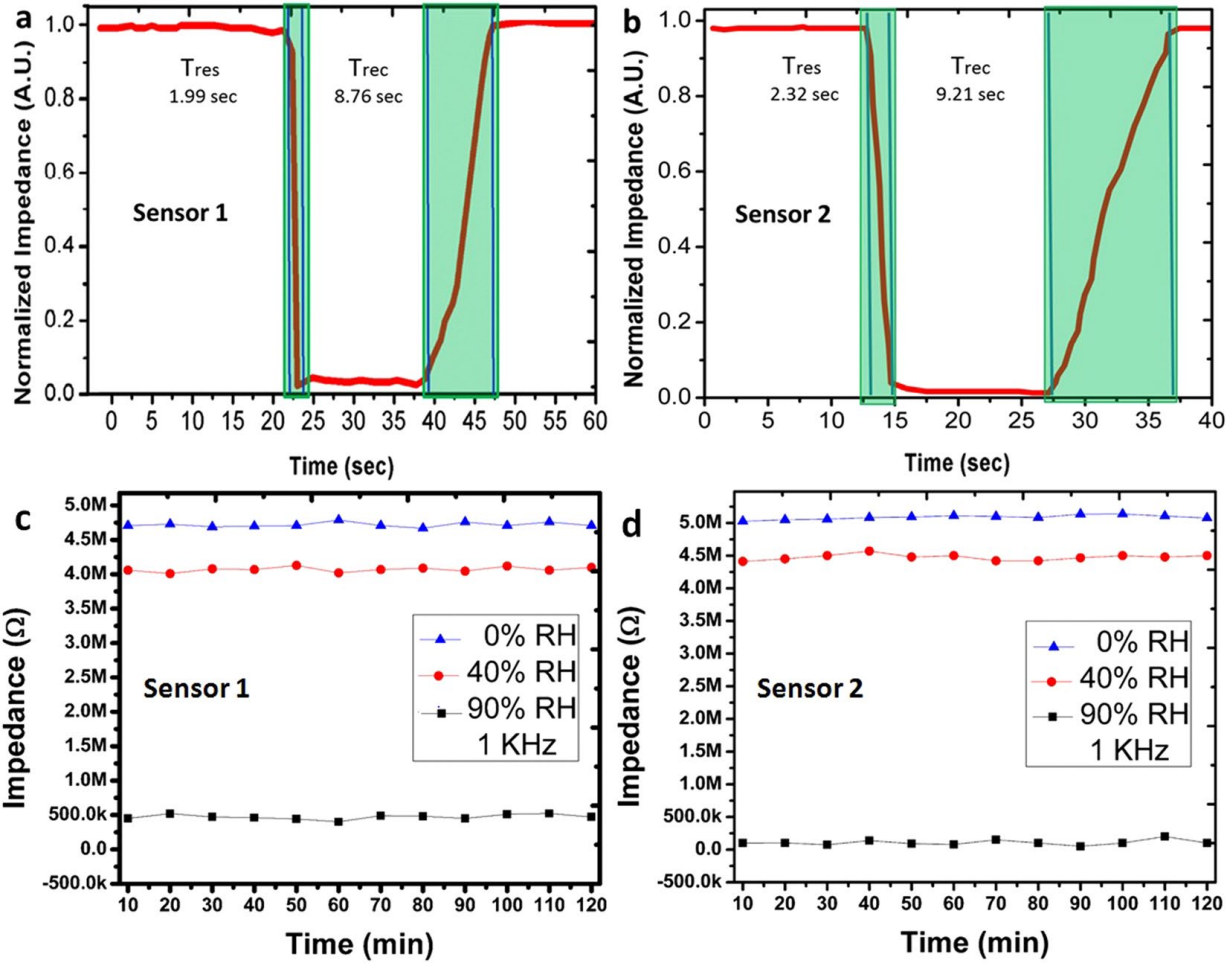
Transient response of (**a**) sensor 1 and (**b**) sensor 2. Stability on 0% RH, 40% RH (open air), and 90% RH of (**c**) sensor 1 and (**d**) sensor 2.

The transient response of SUT is shown in Fig. 8, and it was recorded using breath detection system^9^. The exhaling and inhaling act as humidification and dehumidification, respectively. The transient response of the sensor indicates that, sensor can detect sudden change in humidification levels in environment. The T_res_ and T_rec_ of sensor 1 was recorded as ~1.99 sec and ~8.76 sec, respectively as shown in Fig. 8a. The T_res_ and T_rec_ of the sensor 2 was recorded as ~2.32 sec and ~9.21 sec, respectively as shown in Fig. 8b. The Transient response of the fabricated sensor has fast response time and recovery time and it can be employed in the real life for the humidity sensing purpose. The stability of the fabricated sensor 1 and sensor 2 were investigated at 1 kHz and both sensors were kept in the ambient chamber for 120 min at 90% RH, at 40% RH (open air response) and 0% RH. Both sensors as shown in Fig. 8c,d maintained stable impedance response at 90%, 40%, and 0% RH, respectively. The sensor 1 maintained a stable impedance response with maximum 0.17% error rate as shown in Figs. 8c and 0.198% error rate was recorded in sensor 2 as shown in Fig. 8d.

### Mechanism

**Figure 9:**
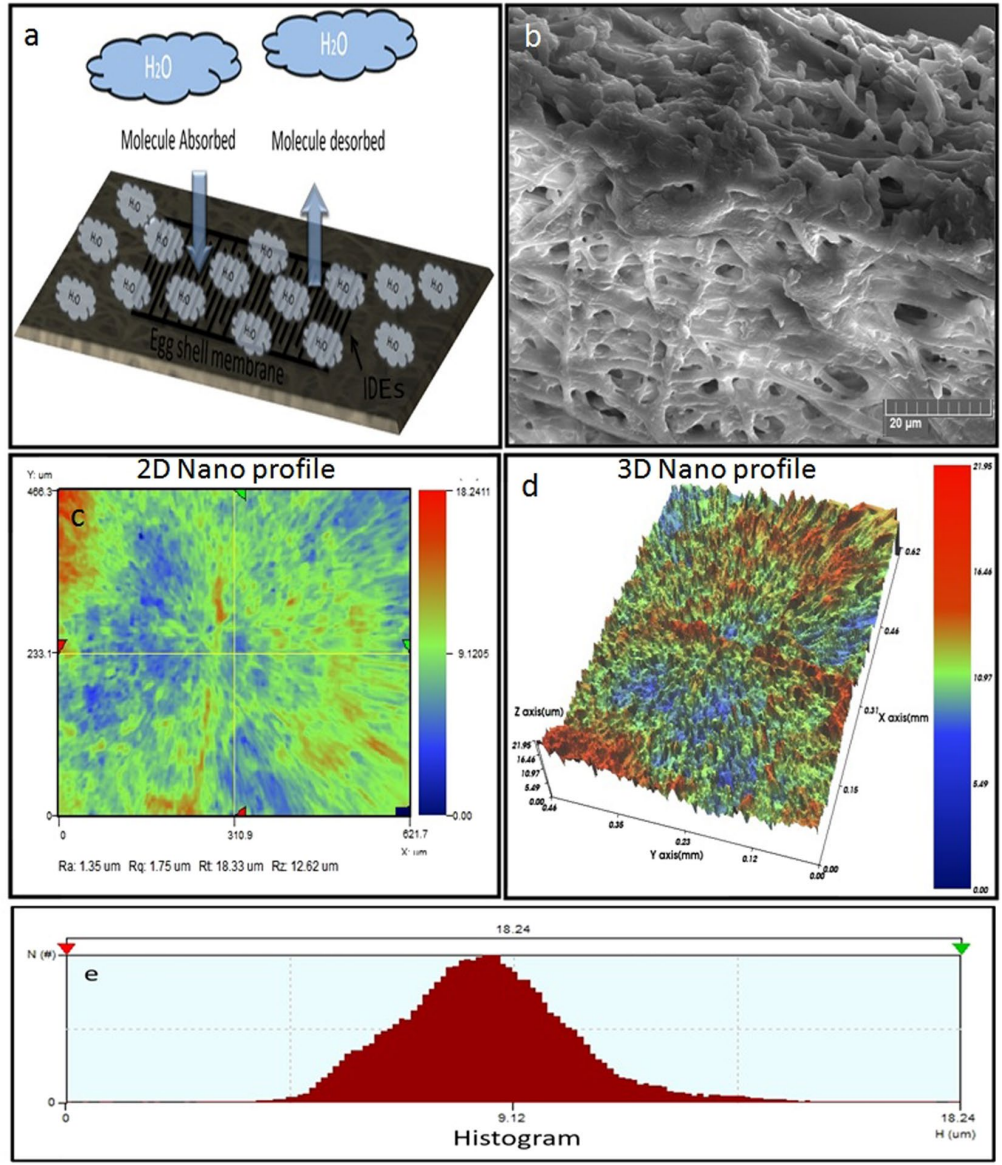
(**a**) Sensing mechanism of the IESM showing adsorption of water molecules and resulting ionic current flow through the thin film. (**b**) Cross sectional image of the IESM at 50 μm magnification, and (**c**) 2D and (**d**) 3D nanoprofile of IESM. (**e**) Histogram of IESM.

The proposed bio-compatible humidity sensor has been reported that the main constituent of the IESM are collagen and saccharides^39^. The chemical composition of the membrane consists of uronic acid and amino acids (alanine and glycine)^40^. The uronic acid is a product of monosaccharide in which the terminal carbon’s hydroxyl group has been oxidized to carboxylic acid. It contains both aldehyde and carboxylic acid moieties and lot of hydroxyl, carbonyl, and amino functional groups^40^ are present on the protein fibers of IESM^39^ to interact with moisture content present in the environment^37^ and can be used as a humidity sensing layer. Hence, the humidity sensing mechanism can be explained as shown in Fig. 9a. It is a porous structure as shown in Fig. 9b, which allows the flow of air and moisture^37^ and it can easily can catch humidity. The structure of IESM also can observed using NV-2000 Universal non-contact surface profiler, which indicate that IESM has surface roughness of 1.35 μm and it also confirm the average thickness between ~18 to 21 μm as shown in Fig. 9c,d. Due to the natural multilayer crosslinked fiber structure of the IESM, its porous structure can be observed using 2D and 3D nano profile as given in Fig. 9c,d. It indicates that IESM is highly porous and consist of micro spacing fibrous structure. The height profile of IESM is shown in Fig. 9e, which indicate that average height of the IESM is 18.24 μm. The IESM is a good insulator and in dried form its impedance value is very high, and the impedance change depends on the water content inside the IESM. The relative permittivity of water significantly differs from the dried IESM. The absorption of water molecules in the thin film of the IESM can dissolve the dried organic materials inside the cells that result in the change in impedance and ionic current flow through the IDEs. From these results, the proposed IESM based humidity sensor shows a notable characteristics comparing another natural materials^21^ for humidity sensing as shown in Table 1.

**Table 1:** Comparison of Egg membrane humidity sensor with onion membrane humidity sensor.

No.	Bio material membrane for humidity sensor	Sensor type	RH range	Response Time	Recovery Time	References
1	Egg shell membrane	IDEs (Impedance and capacitive based sensor)	0–90%	~1.99 sec	~8.76 sec	This work
2	Onion membrane	IDEs (Impedance based sensor)	0–80%	1 sec	10.75 sec	^21^

### Hysteresis curve

**Figure 10:**
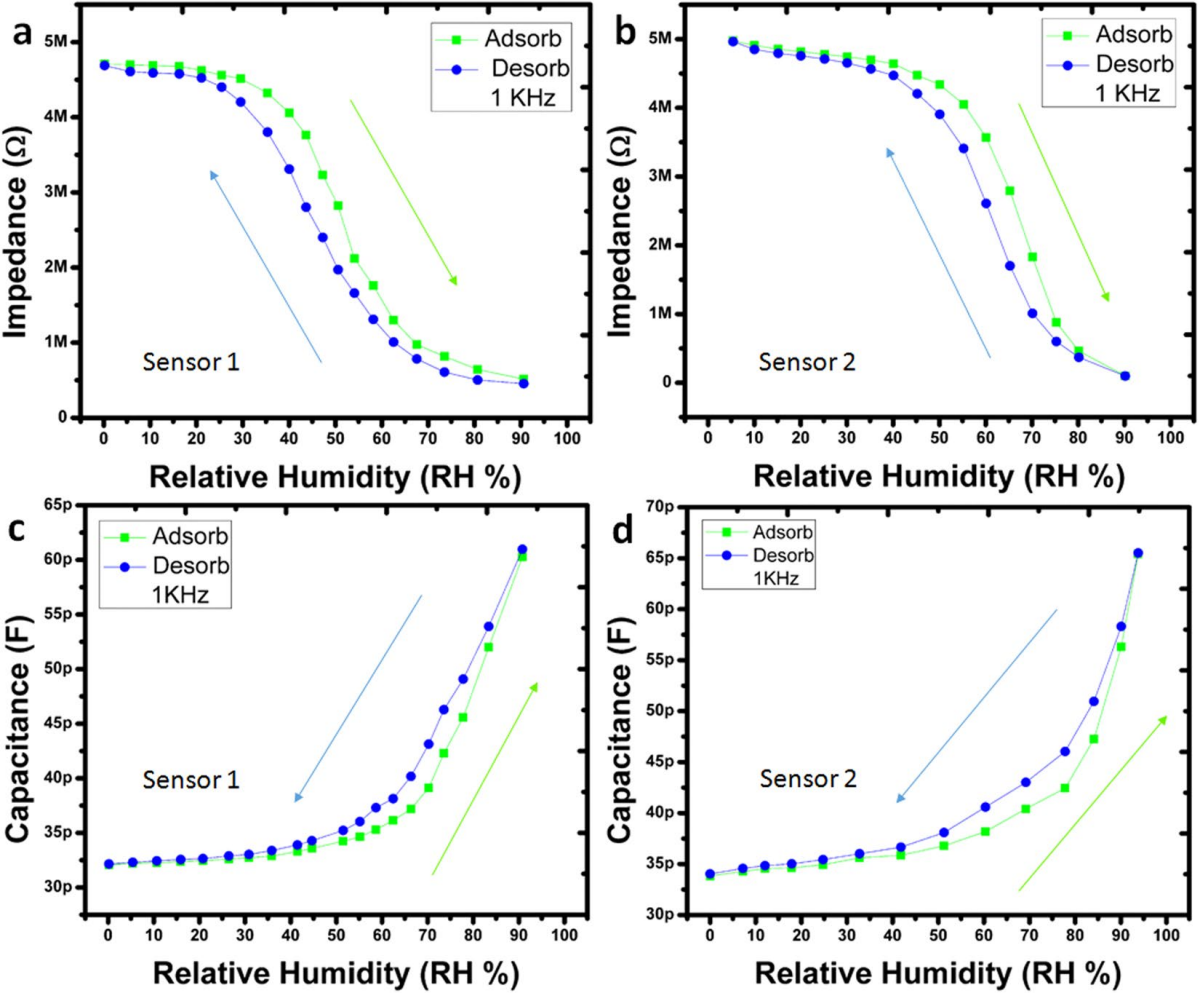
Impedance response hysteresis curve of (**a**) sensor 1 and (**b**) sensor 2 showing abortion and desorption from RH 0–90% at 1 kHz. Capacitance response hysteresis curve of (**c**) sensor 1 and (**d**) sensor 2 showing adsorption and desorption cycle with humidity range from 0 to 90% at 1 kHz.

The main reason of the hysteresis in the IESM is due to the porous structure and chemical absorption. The proposed senor 1 and sensor 2 was analyzed for the hysteresis characteristics at 1 kHz as shown in Fig. 10. Here, sensor 1 and sensor 2 were fabricated with the different size 10 mm × 4 mm and 12 mm × 6 mm, respectively, using inkjet printing technology in Fig. 10. The main reason to observe hysteresis at 1 kHz is, IESM is showing large change in impedance and capacitance at 1 kHz as compare to 10 kHz. For this characteristic, the sensor was sorted at 0% RH and then humidity level was increased to 90% and back from 90% to 0% RH. The impedance and capacitance was recorded against each humidity level during absorption and desorption cycles. The impedance hysteresis is shown in Fig. 10a,b and capacitance hysteresis is shown in Fig. 10c,d of the sensor 1 and sensor 2. In each case hysteresis curve is observed from 30% RH to 80% RH.

## Conclusion

For weather monitoring applications, the paper proposed the bio-compatible humidity sensor based on IESM. This IESM applied as a humidity sensing layer and substrate using IESM. Here, Inter digital electrodes (IDEs) with finger width of 100 μm and spacing of 100 μm were deposited on the surface of IESM through Fujifilm Dimatix DMP 3000 inkjet material printer for sensor 1 and sensor 2. The proposed sensor achieved a good response and recovery time, and its impedance and capacitance change are recorded from 0% RH to 90% RH. Hence, the 19 μm thin IESM could be used to replace flexible and ultra-thin substrate based on PDMS. The Possible areas of IESM were applied as ultra-thin substrate for electronic skin applications, which could be conformably transferred to different arbitrary shape surfaces. The IESM is an environment friendly and can be used as a disposable substrate for environment sensing applications.

## Materials and Methods

### Characterizations

Different characterization techniques are used to investigate the chemical and structure composition of IESM as shown in Figs 2–4 and 9. The SEM has been powerful tool for characterizing fundamental physical properties and surface morphology of the samples. The morphology of IESM can be studied by coating sample with platinum (Pt) sputter using Pt 20 mA 120 mode with Scanning electron microscopy (SEM) Jeol JSM-7600F. The damages may occur on the surface of an IESM during fabrication process. For this reason, the fabricated sensor was observed with optical microscope after each step to make sure that, IESM was intact and IDEs were properly fabricated with the DMP-3000 inkjet printer. 2D and 3D nano profile of top electrode and IESM was analyzed with NV-2000 Universal non-contact surface profiler for roughness measurement in phase shifting minterferometry (PSI) mode. The Fourier transform infrared spectroscopy (FTIR) spectra of an IESM were recorded on a Bruker IFS 66 V spectrometer by using the potassium bromide (KBr) pellet, at a resolution 4 cm^−1^. EDS mapping is performed to confirm the element by element composition of IESM using TESCAN MIRA 3 STEM.

### Materials

Fresh egg was processed using white vinegar. The humidity sensing electrodes were patterned on the surface of the IESM using the Ag nanoparticle ink with 50% dispersion in triethylene glycol monomethyl ether (TGME). Ethanol and de-ionized water was used for cleaning purpose and purchased from sigma Aldrich. The silver (Ag) conductive epoxy CW2400 was used for connecting wires and purchased from circuitsworks. Dry nitrogen gas (N_2_) was used as dehumidification.

### IESM processing

The manual separation of the IESM can be done, but it is difficult to obtain the IESM as it is tightly bonded to the mineral shell. The egg shell is made of calcium carbonate crystals^41,42^. It consist of a porous structure^37^, that is why IESM is called semi permeable membrane^43^. IESM can be easily extracted by dipping the egg in vinegar. The IESM processing is given in Fig. 1a as we dipped the egg in vinegar at room temperature 28 °C. During reaction tiny bubbles of CO_2_ will be released on the surface of vinegar, as a result it will release carbon dioxide (CO_2_)^44^. Then, the naked rubbery egg will remain at the end after 3 days. We make a small hole on the surface of the IESM and pour out the yolk and albumen. The IESM was cleaned with 20 ml ethanol and 30 ml de-ionized water, and place it on a flat surface in a high tension wet state to remove all the water trapped inside the membrane and make it dry at room temperature.

### Sensor fabrication

To fabricate the proposed sensor 1 and sensor 2 on the surface IESM, the IDEs were designed using EAGLE version 7 in dxf format. The design file was converted into bmp file format using ACE 3000 and exported to Dimatix Drop manager software, which converts bmp into ptn format. The ptn file was loaded into software controlled Fujifilm Dimatix DMP-3000 material inkjet printer. The Ag ink was loaded in 10 pL 16 nozzles cartridge with diameter of 9 μm. The 30 V were applied on cartridge nozzles for a stable printing with drop spacing of 20 μm as shown in Fig. 1b and a temperature of the printing platform was controlled at 30 °C through software control system attached with the inkjet printer. IESM was pasted on a flat PET and wrinkles were removed using a tissue paper and it was fixed at its position using tape. The proposed sensor was fabricated by printing IDEs on the surface of IESM through Fujifilm Dimatix DMP-3000 material inkjet printer. The IESM was selected due to good moisture sensitivity, and it can easily detect a small change of humidity. The applied IDEs were designed as electrode finger width of ~100 µm and electrode space of ~100 µm as shown in Fig. 1c, and these parameters were optimized through experimental process to achieve a high sensitivity of the IESM. The IESM were cracked at high temperature annealing due to shrinking of organic nature of thin membrane. That’s why fabricated IDEs are cured at low annealing temperature for longer time duration at 30 °C for 1 hour. The fabrication process of the IDEs on the surface of IESM is explained in detail as given in Fig. 1.

### Humidity analysis

Humidity setup consist of airtight homemade humidity box, HTU21D sensor with resolution 0.04% RH, accuracy ±2% RH, temperature coefficient −0.15% RH/°C and response time <5 sec was used as a reference sensor. Arduino UNO is used as a controlling board and PC is used for data acquisition. The impedance of the fabricated sensor was measured with KEYSIGHT Digital U1700C handled LCR meter, for humidification and dehumidification humidifier and dry nitrogen gas (N_2_) are used, respectively. Cool term software is used for reference sensor data logging and OrignPro 8.0 is used for graph plotting. The fabricated sensor 1 and sensor 2 data is logged with built-in software of KEYSIGHT Digital U1700C handled LCR meter.

**Figure 11:**
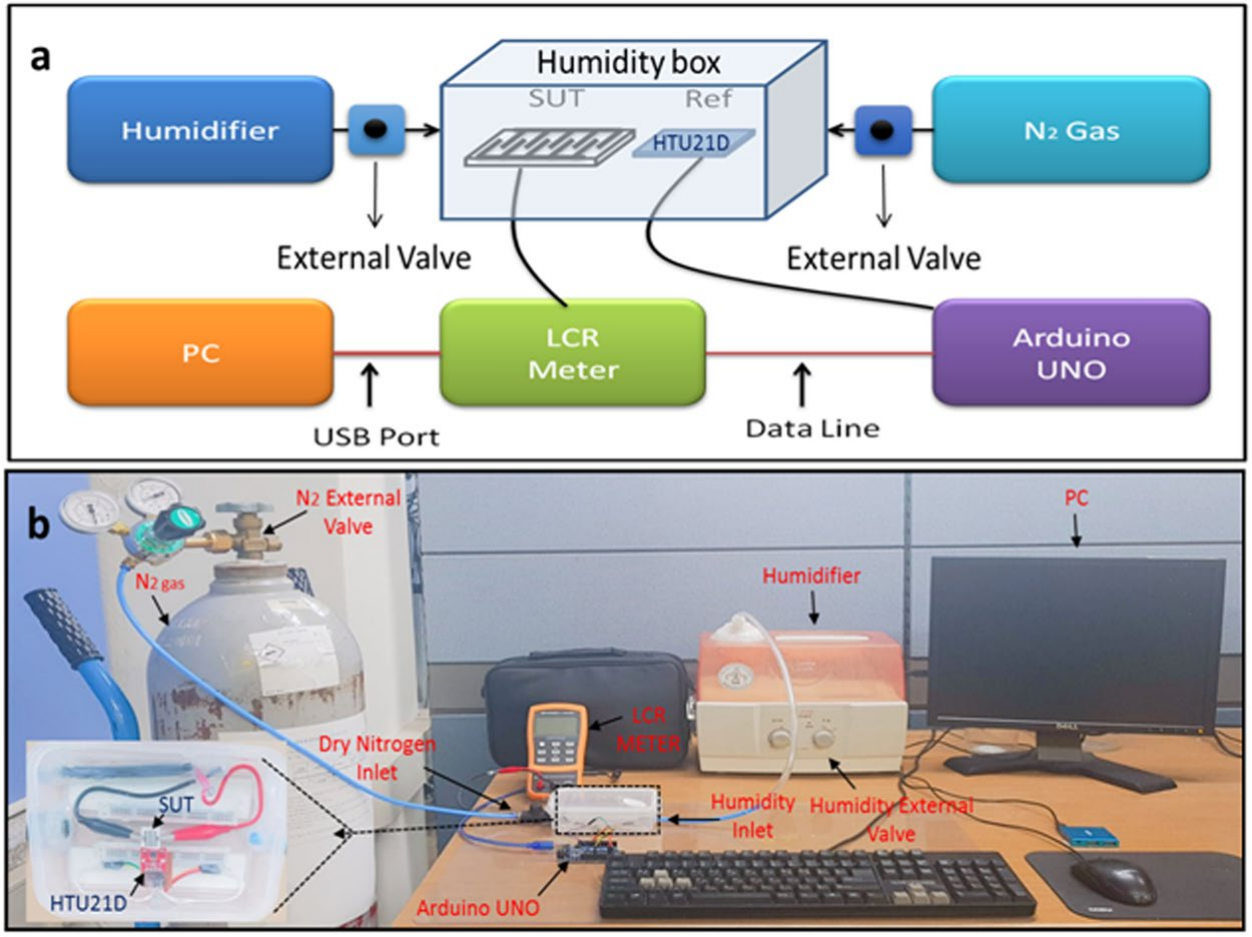
(**a**) Schematic diagram of the humidity verification setup. (**b**) Realized image of the humidity setup using Arduino UNO, DHT21D humidity sensor, sensor under test (SUT), KEYSIGHT LCR meter, humidifier, dry nitrogen (N_2_), LCD, and PC.

Figure 11a shows the block diagram of the humidity setup. The airtight humidity box covers the humidity from 0% to 100% RH respectively with reference sensor and the IESM as a sensing layer and substrate with printed IDEs used as a SUT. The reference sensor is connected with Arduino UNO and SUT is connected with LCR meter. For real time data acquisition, LCR meter and Arduino UNO are connected with PC through data line (USB port) for automatic data logging. The dry N_2_ is injected for dehumidification level from 40% to 0% and the commercialized humidifier is used to increase the humidification level from 0% to 100%. The external valve is used to control the level of humidity inside airtight box using humidifier and N_2_ gas. The temperature of the experiment setup was controlled at 25 °C. Figure 11b shows the realized image of the humidity setup. Using this setup, we measured the proposed sensors. The transient response is measured with sudden increase in humidity level from 0% RH to 100% RH and then dehumidified the sensor from 100% RH to 0% RH.

### Sensor transferred to different curvature surfaces

The targeted surface was wetted with water and non-printed side of IESM was placed on the wet surface as shown in Fig. 6a–c. Due to the fibrous and porous nature of IESM, it absorbs water droplets on the target surface upon coming in contact and it becomes a flexible and soft. After the placement of IESM on an arbitrary surface as substrate, the membrane was dried with blowing hot air. This process removes the trapped water inside the membrane and it is ready to test humidity on transferred substrate.
